# Real-time direct and diffraction X-ray imaging of irregular silicon wafer breakage

**DOI:** 10.1107/S205225251502271X

**Published:** 2016-01-05

**Authors:** Alexander Rack, Mario Scheel, Andreas N. Danilewsky

**Affiliations:** a European Synchrotron Radiation Facility, Grenoble, France; b Synchrotron Soleil, Gif sur Yvette, France; c Kristallographie, Institut für Geo- und Umweltnaturwissenschaften, Albert-Ludwigs Universität Freiburg, Germany

**Keywords:** materials science, X-ray diffraction imaging, phase-contrast X-ray imaging, time-resolved studies

## Abstract

Fracture and breakage of single crystals, particularly of silicon wafers, are multi-scale problems: the crack tip starts propagating on an atomic scale with the breaking of chemical bonds, forms crack fronts through the crystal on the micrometre scale and ends macroscopically in catastrophic wafer shattering. Total wafer breakage is a severe problem for the semiconductor industry, not only during handling but also during temperature treatments, leading to million-dollar costs per annum in a device production line. Knowledge of the relevant dynamics governing perfect cleavage along the {111} or {110} faces, and of the deflection into higher indexed {*hkl*} faces of higher energy, is scarce due to the high velocity of the process. Imaging techniques are commonly limited to depicting only the state of a wafer before the crack and in the final state. This paper presents, for the first time, *in situ* high-speed crack propagation under thermal stress, imaged simultaneously in direct transmission and diffraction X-ray imaging. It shows how the propagating crack tip and the related strain field can be tracked in the phase-contrast and diffracted images, respectively. Movies with a time resolution of microseconds per frame reveal that the strain and crack tip do not propagate continuously or at a constant speed. Jumps in the crack tip position indicate pinning of the crack tip for about 1–2 ms followed by jumps faster than 2–6 m s^−1^, leading to a macroscopically observed average velocity of 0.028–0.055 m s^−1^. The presented results also give a proof of concept that the described X-ray technique is compatible with studying ultra-fast cracks up to the speed of sound.

## Abstract 2

Crack propagation in an Si wafer under thermal stress is depicted by combining diffraction and transmission X-ray imaging. The use of synchrotron radiation allows for the high imaging frame rates required to follow the crack dynamics in real time.

## 1. Introduction   

Wafer breakage during high-temperature processing is a severe problem in semiconductor device manufacturing. Silicon wafers for electronic device production typically contain microcracks at their edges, resulting from mechanical grinding or from handling and shipping. During thermal processing in semiconductor manufacturing, catastrophic total wafer breakage may result from such microcracks and it is an increasingly expensive hazard for production, with costs of millions of dollars per production line per annum (International SEMATECH Manufacturing Initiative, 2004 ▸). Therefore, a basic understanding of crack formation in silicon is an important issue for industrial applications.

Statistical *ex situ* studies of microcracks from nano-indentation reveal that brittle fracture is a stochastic and multi-scale process which is hard to predict even under controlled experimental conditions (Cook, 2006 ▸). The crack tip starts propagating on an atomic scale with the breaking of chemical bonds, forms crack fronts through the crystal on the micrometre scale and ends macroscopically in catastrophic wafer shattering, and this is demanding for theoretical modelling and simulation (Bernstein *et al.*, 2009 ▸). The direct observation of cracks is a challenge because cracks can propagate in silicon with speeds of up to 1500 or 3500 m s^−1^ along the {110} and {111} cleavage planes, respectively (Sherman, 2006 ▸), which are near the speed of sound in silicon. Crack tip simulations on an atomistic level are reported to result in non-mirror-like cleavage surfaces and retardation of the crack tip speed to 800 m s^−1^ (Kermode *et al.*, 2008 ▸). The question arises as to whether even slower macroscopic velocities may result from more complex fracture patterns along high-indexed cleavage faces of higher energy combined with short arrests of the crack front. Such irregular fracturing may result in catastrophic wafer shattering into many small irregular pieces, instead of cleaving into two pieces with smooth {110} faces (Tanner *et al.*, 2015 ▸). For both cases the crack front starts from a failure point if the critical strain is exceeded by *e.g.* external thermal stresses during temperature treatment (Sherman, 2006 ▸; Danilewsky *et al.*, 2013 ▸).

Both long- and short-range strains in crystals are well suited to study using X-ray diffraction imaging (topography) with a white beam (Chikawa, 1968 ▸; Tuomi *et al.*, 1974 ▸; Hartmann *et al.*, 1975 ▸). The technique benefits from the use of hard and intense synchrotron radiation, which has already allowed the *in situ* study of the much slower dislocation dynamics in silicon wafers with a reasonable temporal resolution of about 1 s (Tuomi *et al.*, 1983 ▸; Danilewsky *et al.*, 2011 ▸).

Our own *in situ* experiments showed that, during processing at high temperatures above the brittle-to-ductile transition, slip bands nucleate from strained areas around microcracks from preparation (Danilewsky *et al.*, 2011 ▸). Once thermal slips are formed, cracks do not develop any more (Danilewsky *et al.*, 2013 ▸). In the brittle regime, fracture happens only from critical cracks. In earlier work (Tanner *et al.*, 2012 ▸) it was concluded that the aspect ratio κ of strain-related contrast in diffraction images around a crack is a reliable measure of wafer fracture. κ is the crack length *L* divided by the width of the contrast *d* at the tip: κ = *L*/*d*. A large κ value increases the possibility of catastrophic wafer fracture during wafer handling (the dimensionless aspect ratio κ is – unlike *e.g.* polarizing infrared microscopy – widely independent of the sample or experimental properties) (Cook, 2006 ▸; Tanner *et al.*, 2012 ▸). However, because of a frame rate of one image every 0.72 s, only a minimum crack tip velocity of 7 × 10^−3^ m s^−1^ could be determined for a critical crack which appeared between two frames, propagating in the {110} plane in the 〈110〉 direction (Danilewsky *et al.*, 2013 ▸).

Unprecedented temporal resolution with hard X-ray imaging can be reached at synchrotron light sources thanks to high-speed CMOS cameras used in combination with polychromatic illumination (Rack *et al.*, 2010 ▸). Recently, with exposure times short enough to exploit the pulsed time-structure of the beam produced by the European Synchrotron Radiation Facility (ESRF), mechanically induced cracks in glass could be depicted by so-called single-bunch imaging (Rack *et al.*, 2014 ▸), a technique originally pioneered at the Advanced Photon Source (Argonne National Laboratory, USA) (Luo *et al.*, 2012 ▸).

Diffraction imaging reveals information about the strain conditions and strain release around a crack. To establish its shape and orientation a direct image is essential. A basic understanding of the dynamics of silicon breakage requires information obtained by synchronizing the two imaging methods. Hence, combining direct transmission imaging with X-ray diffraction topography using high-speed detectors at synchrotron light sources can open a path towards understanding crack propagation in silicon wafers.

## 2. Experimental   

**Figure 1:**
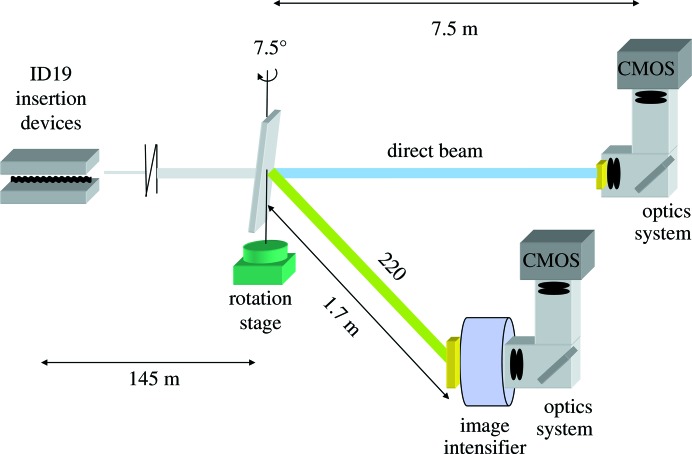
A sketch of the real-time diffraction topography imaging setup. White radiation from two undulators impinges on a silicon wafer. Both the 220 reflection topograph and the direct transmission image are recorded simultaneously. Both imaging detectors are equipped with a high-speed camera in order to allow for a short exposure time (1.28 µs) and a high image-acquisition rate (∼35 500 images per second).

For the first experiments of this kind, beamline ID19 of the ESRF was chosen as a large (white) beam of up to 5 × 1.5 cm is accessible (Weitkamp *et al.*, 2010 ▸). Furthermore, the beamline can be operated with a minimal number of optical elements (here, a 1.4 mm-thick diamond absorber and one Be window) in the X-ray beam path, which ensures high sensitivity for topographic and phase-contrast imaging (Espeso *et al.*, 1998 ▸). The experiments were carried out while the ESRF operated in the so called four-bunch mode, where only four highly populated electron bunches are used in the storage ring, separated from each other by a temporal delay of around 700 ns. A sketch of the experimental setup is shown in Fig. 1 ▸. A silicon single crystal of dimensions approximately 10 × 20 mm was illuminated by the filtered white beam (15 × 8 mm, energy spread from around 14 to 40 keV) of the beamline’s two u32 undulator insertion devices (gaps 11.73 and 11.92 mm, tuned for maximum reflected intensity at the angle described more fully below, *i.e.* the first harmonic transmitted by the filters was trimmed at around 25 keV and the bandwidth of the harmonic was approximately 2%).

The silicon crystal was placed at an angle of about 7.5° with respect to the incoming beam in order that the 220 reflection diffracts radiation at approximately 25 keV onto a large-area detector consisting of a 200 µm phosphor screen at input (CsI:Na), an image intensifier with a P46 (YAG:Ce) phosphor screen at output, visible light optics and a CMOS camera [type pco.Dimax (PCO AG, Germany), no synchronization with the radio frequency of the storage ring] – the combination of fast scintillators ensures the absence of ghost images at the desired high frame rates (Ponchut, 2001 ▸). The detector was positioned around 1.7 m downstream of the crystal and recorded topographs in Laue geometry with an exposure time of 1.28 µs at an acquisition rate of 35 511 images per second (nominal pixel size 62 µm). A second detector approximately 7.5 m downstream (250 µm-thick LuAG:Ce single-crystal scintillator lens coupled to a pco.Dimax camera, nominal pixel size 40 µm, well adapted to the large propagation distance, exposure time 1.28 µs at an acquisition rate of 35 504 images per second; only this system was synchronized with the radio frequency of the storage ring) recorded direct transmission images in inline X-ray phase-contrast mode (Cloetens *et al.*, 1996 ▸; Rack *et al.*, 2014 ▸). Penumbral blurring in the direct transmission images can be neglected, thanks to the long distance between the source and the experimental station of about 150 m (Cloetens *et al.*, 1996 ▸; Espeso *et al.*, 1998 ▸). The exposure time of 1.28 µs is short enough that the recorded images are dominated by the flash of light from a single bunch, *i.e.* with respect to the recorded intensities shown the setup is already compatible with single-bunch imaging (Rack *et al.*, 2014 ▸). The detection limit for the crack tip in direct transmission can be roughly estimated from the detector parameters used. The crack tip itself is not directly resolved but is detected *via* a phase-contrast related fringe, due to the large propagation distance of 7.5 m between the wafer and detector (Cloetens *et al.*, 1996 ▸). Due to the 40 µm pixel size of the transmission image detector, and knowing that phase contrast can be exploited to detect features up to two orders of magnitude smaller than the resolving power of the detector (Zabler *et al.*, 2010 ▸), the crack is only visible once it has an opening displacement of at least several hundred nanometres. Note that the direct image detector operates in the edge-enhancement regime, with the pixel size being substantially larger than the width of the first Fresnel zone of (λ*z*)^1/2^ ≃ 19 µm [with λ the (mean) wavelength seen by the detector and *z* the propagation distance].

In order to create reproducible starting conditions for the cracks, standard (001) silicon wafers were damaged artificially using the nano-indentation technique (Garagorri *et al.*, 2010 ▸). A Vickers tip at a load of about 50 N was applied. In order to induce thermal stresses and initiate cracking from an indent, the silicon wafer was heated up to about 1000°C by a gas burner (therefore the heat load to the sample introduced by the impinging radiation can be neglected) and then quenched with a water jet while the movies were acquired. The increase of strain fields in a hot silicon wafer induced by water droplets is resolved from the moment before new cracks nucleate until propagation stops. As already shown in earlier work (Tanner *et al.*, 2012 ▸, 2015 ▸), cracks arise and propagate during heating/cooling along the border between compressive and tensile strained areas. As a result, curved irregular (*hkl*) crack faces occur, rather than perfect mirror-like {110} or {111} cleavage planes.

## 3. Results and discussion   

**Figure 2:**
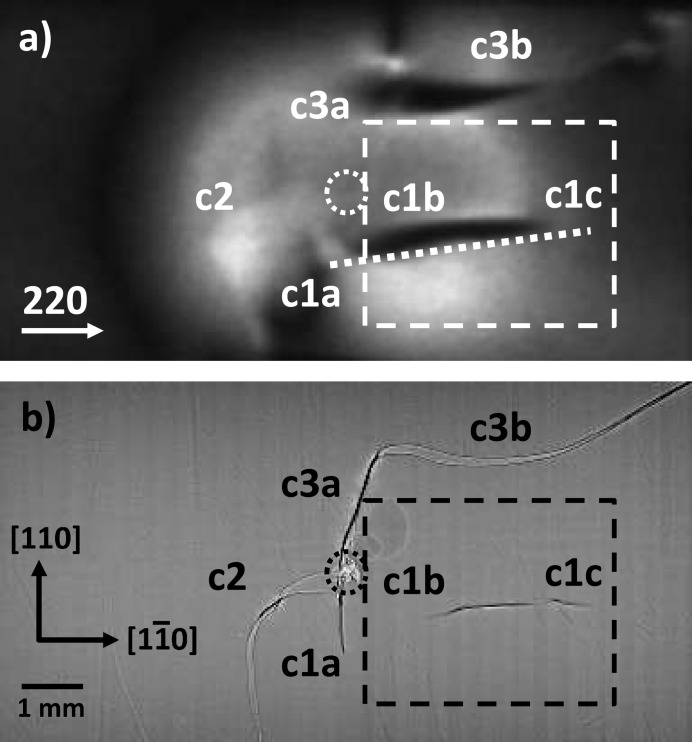
An (001) Si wafer with Vickers indent (dotted circles) at the centre. The edges correspond to the <110> directions. (*a*) The 220 diffraction image with all the cracks, *i.e.* the final stage (frame No. 3000 of the acquired movie with 1.28 µs exposure time; the diffracted image is scaled and rotated to match the transmission image). The inclined dashed line marks the position of the intensity profiles shown in Fig. 4 ▸. (*b*) The sum of 100 direct images acquired, showing the cracks in their final state. At the centre of the image the indent is visible. The segments of three cracks are labelled c1a–c3b. In both parts, the dashed boxes mark the area selected by way of example for the time series shown in Fig. 3 ▸.

Before the heating and quenching process, the indents at the centre or near the edge of the Si wafers are clearly visible in both the direct and the diffracted images (*cf.* Fig. 2 ▸). During heating and quenching, strain-induced contrasts increase around the indent until the first crack nucleates and reduces the strain, which is only visible in the diffraction image.

The overview in Fig. 2 ▸ shows the discussed example, sample No. 5. All three cracks identified nucleate at the indent, as expected, and are named in both pictures as cracks c1–c3 with single segments a–c. All the cracks are curved and they do not follow the expected 〈110〉 cleavage directions, which may be the result of a non-homogenous and curved temperature field (Tanner *et al.*, 2015 ▸). Fig. 2 ▸(*a*) shows X-ray diffraction image No. 3000 from the acquired movie and Fig. 2 ▸(*b*) shows the sum of the direct images, *i.e.* the final stage of the cracks. The dashed box in Fig. 2 ▸(*b*) indicates the area which was selected for a more detailed analysis of the crack propagation.

**Figure 3:**
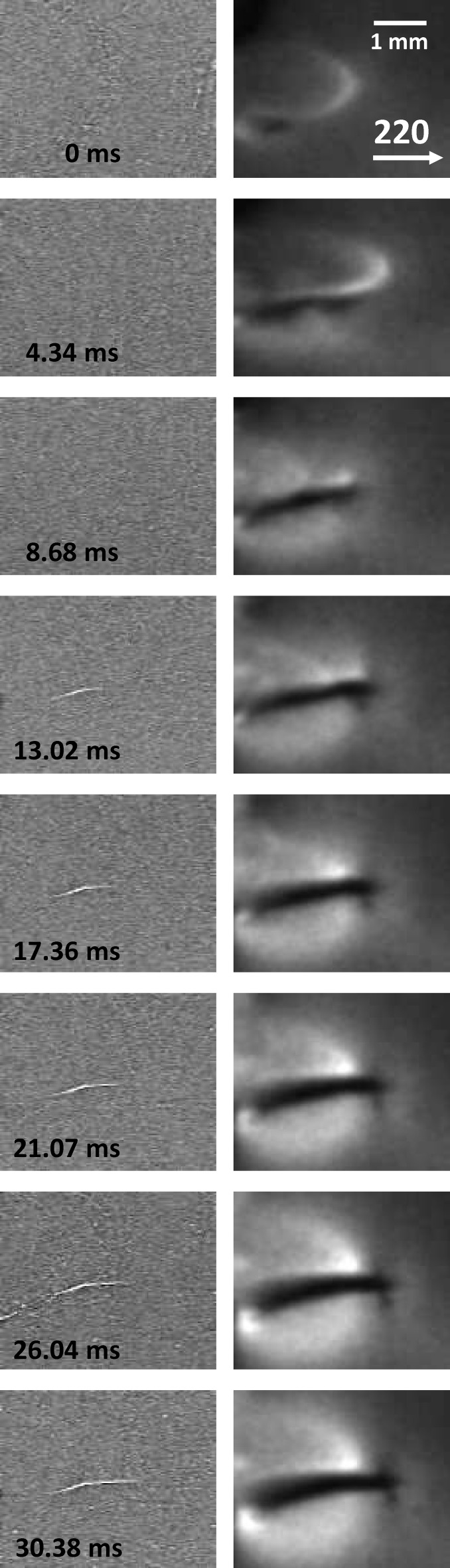
Selected images from a series of 3000 showing crack propagation in a silicon wafer under thermal stress (compare crack c1c in Fig. 2 ▸). (Left) The direct transmission images. (Right) The diffraction images with the 220 reflection (the diffraction images have been scaled and rotated to match the transmission images).

From that region of interest, Fig. 3 ▸ shows selected direct transmission and diffraction images of the relatively slow-growing crack c1. Segment c1a nucleates at the indent, which is outside the region of interest. The inclined part c1b deflects into the horizontal part c1c which runs, at the front surface of the wafer, perfectly parallel to 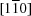. The images are a subset of the acquired movie which consists of 3000 images (according to the acquisition rate of 35 511 images per second, the temporal spacing between two frames is 28.1 µs; a subset of 1250 images which covers the dynamics discussed in this paper is available as a .avi file in the supporting information). The first image shown is taken approximately 7 ms after the first water droplets hit the hot wafer, *i.e.* thermal stress is already present. Single water droplets and water wavefronts are visible in the direct transmission images. For the movie, time 0 is defined from the moment the crack segment c1a deflects into the inclined segment c1b and arrives in the field of view. The 220 reflection shakes slightly due to vibrations introduced by the rinsing water on the wafer, causing the reflection to move on the detector. A simple median covering three frames was applied along the time axis in order to reduce noise in the direct transmission images.

The macroscopic propagation velocity of the crack tip in segment c1c was measured from the direct images. In the first part of c1c a mean velocity of about 0.055 m s^−1^ results, which slows down abruptly to 0.028 m s^−1^. The complex fracture planes involved are high-index high-energy interfaces of random {*hkl*}, which will be discussed in detail later. These observed velocity values are orders of magnitude slower than expected from the literature for crack surfaces following perfect cleavage planes (Sherman, 2006 ▸).

**Figure 4:**
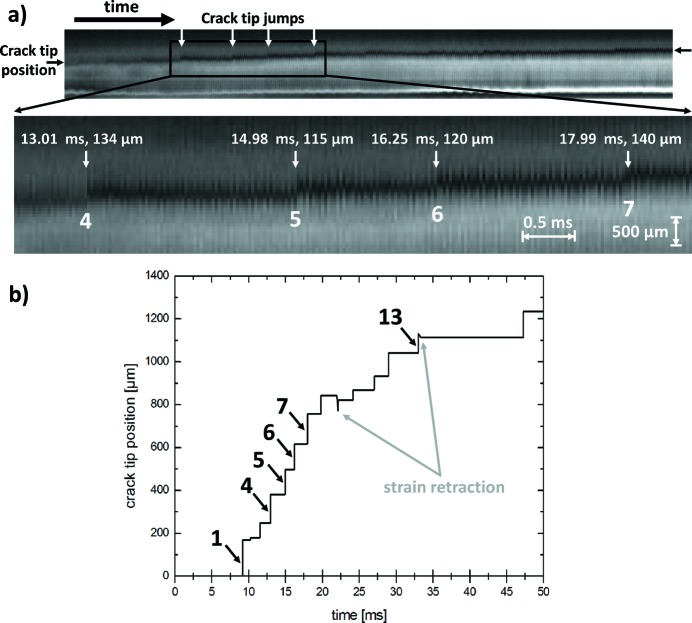
(*a*) Propagation of the crack tip as derived from intensity profiles along the inclined dashed line marked in Fig. 2 ▸(*a*) for all frames of the movie related to Fig. 3 ▸. The position of the crack tip can be tracked indirectly *via* the marked black region, as the tip changes the grey levels locally while travelling. The outlined box and enlargement show by way of illustration a region where the crack propagates in jumps. Position and length are given for jump Nos. 4–7. (*b*) A plot of the crack tip position *versus* recording time reveals sudden jumps in the position during crack tip propagation (*cf.* Fig. 3 ▸, crack segment c1c). Strain retraction occurs at positions where the shape of the crack changes (compare Fig. 5 ▸). The vibration contribution to the crack tip position has been removed.

Further details of the dynamics of the propagation of crack c1 are revealed when looking at intensity profiles acquired along the inclined dashed line in Fig. 2 ▸(*a*): the position of the crack tip is accessible indirectly, *i. e.* by the local change in the grey-level intensity of the strain-related contrast while the crack tip propagates parallel to the dashed line. In Fig. 4 ▸ these profiles are plotted as a function of time for all frames of the movie related to the pictures shown in Fig. 3 ▸, starting with the frame where the first indication of a crack appears. The position of the crack tip is associated with the marked black region. Two features are visible immediately: (i) high-frequency oscillations due to vibrations of the wafer related to the impinging water and (ii) strong discontinuities (four of the 13 are selected for illustration and marked with white arrows). These discontinuities clearly indicate that the crack does not propagate with constant velocity. Hence, the mean slow speed measured results from pinning and reinitiating of the fast propagation of the crack tip.

The 13 jumps observed in the field of view are indicated in Fig. 4 ▸(*b*), as are two positions at which the strain field recedes slightly before reinitiating with a slower mean velocity. The maximum crack tip speeds are higher than can be resolved with the acquisition rate used. The macroscopic velocities reported above are therefore mean velocities. The true values of the velocity between two acquired frames are still beyond the time resolution of the current setup, since the time duration of a jump is less than the time between two frames. The time between two frames is 28.1 µs, so the velocity of events with a duration less than this cannot be measured.

Fig. 4 ▸ shows clearly that there is no continuous crack propagation speed, rather there are jumps with a crack tip speed faster than 6 m s^−1^ followed by stop times of about 1–2 ms. In Fig. 4 ▸(*b*) the crack tip positions are plotted against time. The first jump, related to crack segment c1b, advances by the longest increment of 168 µm. Assuming the maximum speed reported in the literature of 3500 m s^−1^, the first jump would be composed of a 4.8100 × 10^−8^ s movement followed by a 2.8152 × 10^−5^ s stop within the first to second frame time span, continuing during the following 37 frames for about 1.04 ms in total. This high-speed movement followed by a stop of 1–2 ms repeats eight times in the first part of the field of view. After a short strain retraction, the following five jumps are shorter in distance at slightly longer pinning times, resulting in the slow average speed. The mean velocities of 0.055 and 0.028 m s^−1^, respectively, correspond well with the values measured from the direct projection images in phase-contrast mode.

**Figure 5:**
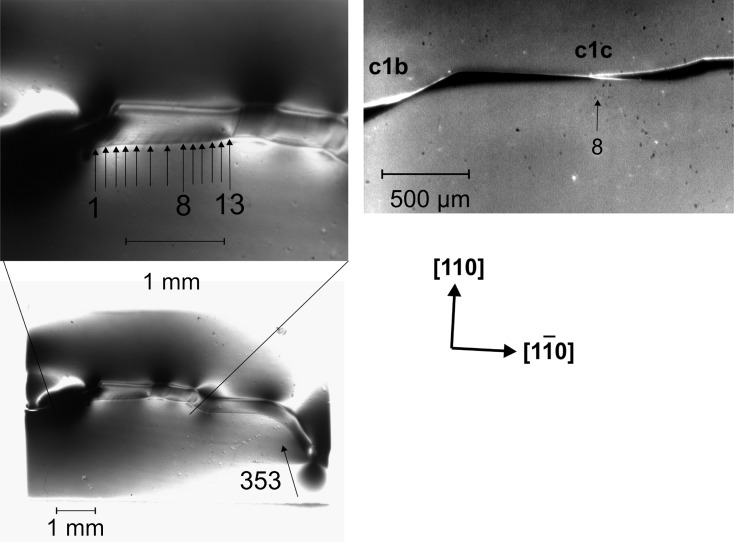
(Left) White-beam topography of crack segment c1c (large-area transmission, high-resolution photographic film), showing lines at the cleavage plane related to the 13 crack tip jumps shown in Fig. 4 ▸(*b*) (indicated by black arrows). (Right) The same crack segment in infrared transmission bright field. At approximately jump No. 8, the crack segment c1c deflects from 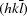 to (*hkl*) with the opposite sense of inclination.

Even if the 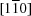 direction of the crack propagation at the front face of the wafer seems to be macroscopically constant, the microscopic surfaces vary and deviate from {110}, and no smooth {110} cleavage plane can be observed. As a result there is a curved trace of the crack at the back face of the wafer (see Fig. 5 ▸). The related shape of the strain field remains more or less constant and increases in size proportionally with the increasing crack length. Besides such a complex fracturing, the broad strain field at the crack tip, which is clearly visible in the diffraction images, leads to small κ values (1 at the beginning and 2 at the end), making this crack a non-dangerous one in terms of wafer breakage. This, in combination with the fact that the crack leaves a cooled region, *i.e.* enters a direction with smaller gradients and strain, prevents full wafer fracture and may be the reason for the observed slow average tip velocity and the fact that the sample is not broken completely.

The irregular behaviour of the crack face formation and propagation can be correlated with features observed in white-beam X-ray transmission topographs taken after the experiment at the TopoTomo beamline at the ANKA synchrotron light source (KIT, Karlsruhe, Germany) using high-resolution photographic film (Rack *et al.*, 2009 ▸). Fig. 5 ▸ (left) shows crack c1b in the highly asymmetric 353 reflection. Due to the chosen projection direction, the crack plane becomes visible in detail from one wafer surface to the other. Extended black contrasts indicate residual strain at some positions along the crack. Whereas the trace of the crack on the front face is a straight line parallel to 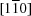, the back face is curved. This is also clearly visible in the infrared transmission micrograph in Fig. 5 ▸ (right). The view, perpendicular to the (001) surface, shows the slightly and continuously changing inclination of the fracture plane. From the width of the cracks and the wafer thickness of 750 µm, the crack faces can be calculated. It starts in this example with 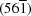 to become (110) around the position related to jump No. 8, and then changes into the opposite inclined (561) face.

The 13 smooth areas, indicated in Fig. 5 ▸ (left) with black arrows, are separated by black lines. Such contrasts arise from micro cleavage steps (mainly along the {110} and {111} planes from the front to the back faces of the wafer), which are often observed on non-smooth cleavage planes (Kaufman & Forty, 1986 ▸) and here are obviously related to the arrests of the crack front (Gleizer & Sherman, 2014 ▸). The crack front may be reinitiated if enough energy for the formation of such a misalignment step is accumulated, and the crack tip then jumps to the next arrest.

The pinning of the crack front between jump Nos. 8 and 9 shows another anomaly. The strain-related contrast reduces slightly before continuing with the already mentioned slower average propagation speed. Because the crack opening is non-reversible, and the stress gradient can be assumed to remain constant, a reduction in the sphere of deformed lattice planes around the crack tip at constant strain means a reduction of stored energy, which can be supposed to be transferred into the crack tip itself. This energy may be needed for the deflection into the opposite inclined crack face behind jump No. 8. The slower average crack speed can be explained with the altered orientation of the cleavage steps on the opposite inclined cleavage plane.

It is easy to see that such irregular radial cracks produce many small splinters, instead of two pieces in the case of a perfect smooth cleavage if the crystal breaks completely.

## 4. Summary and outlook   

Using single-bunch imaging with two high-speed detector systems is a significant step towards genuinely ‘real-time’ imaging and the *in situ* study of processes in the picosecond range. Studying cleavage and fracture in single crystals becomes possible in a combined direct and diffraction mode, and the power of such high-speed imaging has been demonstrated by the example of silicon wafer fracture. We could follow cracks in silicon *in situ* under thermal stress which did not cause complete breakage of the Si crystal. The cracks propagate with what seems at first glance to be a surprisingly slow average propagation rate, with crack fronts which do not follow the perfect {111} or {110} cleavage of the diamond structure. Indeed, the slow average velocity is a consequence of the fact that the crack does not propagate in a continuous manner but rather by sudden and discrete jumps: the average slow velocity results from fast crack propagation, higher than the actual resolution limit of 6 m s^−1^ for a crack tip jump (given by the spatio-temporal sampling as well as the fact that here jumps happen between two frames and two pixels), followed by pinning for 1–2 ms. The arrests correspond to micro cleavage steps on the macroscopic (*hkl*) cleavage planes, which could be resolved by high-resolution X-ray topography after the *in situ* experiment. The reason for such arrests, referred to in the literature as lattice trapping (Bernstein & Hess, 2003 ▸), is still unknown but now becomes experimentally accessible with our high-speed imaging approach.

The true crack velocity of up to the speed of sound could be subject to more detailed studies with an even higher time resolution: the main limitation of our current study is of a technical nature. Replacing the CMOS camera used here with a faster model would allow the time between frames to be reduced to the same order as the exposure time. The exposure times and hence the recorded intensities would remain similar. An example would be the HPV-X2 camera (Shimadzu Corporation, Japan): based on a frame-transfer CMOS concept, this camera can operate at frame rates of up to 10 000 000 images per second, which with the given pixel size would allow for a satio temporal resolution of 2000 m s^−1^ and more (the camera has already been successfully tested as part of another experiment at beamline ID19). Furthermore, with the current construction of so-called diffraction-limited synchrotron light sources, a major gain in performance in terms of photon flux density can be expected within the next few years. The promised increase in brilliance of two orders of magnitude would allow for a drastic improvment in the spatio-temporal resolution, especially for X-ray diffraction topography (Rack *et al.*, 2014 ▸). Hence, more details of the dynamics around the propagating crack tip might become accessible (Dürig & Zimanowski, 2012 ▸). The higher photon flux density will also be available at shorter wavelengths and therefore denser materials such as GaAs wafers could be studied too. In particular, for cracks propagating at higher velocity, exploiting the bunch structure of the electrons in the storage ring will become crucial (Rack *et al.*, 2014 ▸). We are only at the beginning of studying ultra-fast crack propagation in single-crystalline materials in real time.

## Supplementary Material

AVI of the movie shown in Fig 3. DOI: 10.1107/S205225251502271X/ro5006sup1.avi
